# The Phenomenology of Hair Pulling Urges in Trichotillomania: A Comparative Approach

**DOI:** 10.3389/fpsyg.2016.00199

**Published:** 2016-02-19

**Authors:** Shai Madjar, Chandra S. Sripada

**Affiliations:** ^1^University of Michigan Medical SchoolAnn Arbor, MI, USA; ^2^Department of Philosophy, University of MichiganAnn Arbor, MI, USA; ^3^Department of Psychiatry, University of MichiganAnn Arbor, MI, USA

**Keywords:** trichotillomania, hair pulling, phenomenology, urges, resistibility

## Abstract

Trichotillomania is a disorder characterized by recurrent urges to pull out one's hair, but the experiential characteristics of hair pulling urges are poorly understood. This study used a comparative approach to understand the subjective phenomenology of hair pulling: participants with trichotillomania symptoms were asked about their hair pulling urges as well as their urges to eat unhealthy foods. Participants who reported experiencing problematic unhealthy food urges were identified and asked to compare the phenomenological characteristics of their hair pulling and unhealthy food urges across a variety of dimensions. Results revealed significant differences for only some urge properties measured, and differences that existed were small to moderate in magnitude. Qualitative comparisons of the two urges revealed situational characteristics of hair pulling that could explain these small to moderate differences between the two urges. We conclude that hair pulling urges may be more comparable to ordinary urges such as unhealthy food urges than one might expect, but that hair pulling urges may nevertheless be rated as slightly more severe due to situational characteristics of these urges. This conception may improve clinician and lay understanding of the condition, assist with destigmatization efforts, and facilitate the development of treatment strategies.

## Introduction

Trichotillomania (Hair-Pulling Disorder) is a condition characterized by recurrent urges to pull out one's hair, repeated attempts to stop or decrease hair pulling, and significant distress or life impairment (American Psychiatric Association, 2013). The prevalence of this disorder is recognized to be quite high, about 1–2% of the population (Christenson et al., 1991b; American Psychiatric Association, 2013).

Research on trichotillomania has successfully highlighted many important behavioral characteristics of the disorder, including various cues that trigger hair pulling urges; rituals associated with hair pulling (e.g., trichophagy); affective changes associated with hair pulling; and different styles of hair pulling, which include an unconscious and “automatic” type and a conscious or “focused” type (Christenson et al., 1991a, 1993; du Toit et al., 2001; Diefenbach et al., 2002; Woods et al., 2006; Flessner et al., 2008; Grant and Odlaug, 2008; Duke et al., 2009a,b; Shusterman et al., 2009). This body of research has led to the development of cognitive-behavioral models of trichotillomania (for a review, see Roberts et al., 2013). According to these models, hair pulling urges may be triggered and facilitated by various internal and external cues, including hair-pulling implements, mirrors, particular settings, certain visual or tactile stimuli, and cognitive or affective states (Mansueto et al., 1997). Hair pulling urges may then be reinforced, either positively or negatively, by the pleasure, satisfaction, or relief that follows hair pulling (Mansueto et al., 1997; Roberts et al., 2013).

To date, however, the phenomenological experience of trichotillomania sufferers, and in particular the subjective experience of the motivational “pull” of hair pulling urges and the personal sense of controllability over urges, has not been well characterized. This gap in the literature is notable, since so long as the lived experience of trichotillomania remains poorly described, there will be steep barriers to empathic understanding by family, clinicians, and the wider public.

One potentially effective way of studying the subjective experience of trichotillomania urges is to characterize them in direct relation to everyday urges, such as urges to eat unhealthy foods. This method offers the advantage of anchoring the knowledge gained about the phenomenology of trichotillomania in a familiar experience that is widely shared. For example, it would be possible to say that urges in trichotillomania are like a certain class of ordinary urges with respect to certain attributes (e.g., frequency or intensity), but that they differ with respect to other attributes (e.g., automaticity, resistibility, or profile of associated affective states). This comparative approach, we believe, is more illuminating and yields greater understanding of the lived experience of trichotillomania than the alternative approach that lacks a comparator.

Apart from deepening our understanding of the phenomenology of hair pulling urges, an examination of the relationship between hair pulling urges and ordinary urges is important for three further reasons. First, it is widely believed that the therapeutic alliance between patient and clinician is an important determinant of therapeutic outcome, and this view is supported by the data (Martin et al., 2000). A deeper understanding by the clinician of what it feels like to experience hair pulling urges would plausibly increase clinician empathy, strengthen the therapeutic alliance, and thereby improve treatment outcomes. Second, a comparison between hair pulling urges and everyday urges may help to destigmatize a condition that is unfortunately characterized by shame and secrecy. Comparing hair pulling urges to urges that are familiar to the lay public will aid in the articulation of the severity of these urges and may make such urges more understandable. Finally, comparison to ordinary urges could serve as a tool for the identification of any unique characteristics of hair pulling urges that may not have been explicitly recognized to date. Alternatively, similarities between hair pulling urges and everyday urges may spark research interest in adapting techniques and theories about the regulation of ordinary urges for use in addressing hair pulling urges.

From a methodological standpoint, we recognized that it would be difficult for participants to make comparisons between hair pulling urges and the large and heterogeneous class of everyday urges taken together. We therefore sought to identify a specific everyday urge for comparison that would meet the following criteria: (1) The urge should be very common, so that participants with trichotillomania symptoms recruited to the study would be likely to experience such urges on a regular basis; (2) The urge should be such that participants would frequently want to resist it, so that comparison of the resistibility of the urge to the resistibility of hair pulling urges would be straightforward; and (3) The urge should have a comparable temporal profile to a hair pulling cycle. For example, the urge to sleep is a poor candidate for comparison, since sleep is a process that is normally of significantly greater duration than hair pulling.

In a comprehensive study on the occurrence of everyday desires, Hofmann et al. (2012) identified the desire to eat as by far the most common desire on a daily basis. Furthermore, it was found that desires to be fit and healthy were some of the most frequently cited goals that participants identified as being in conflict with their ordinary desires. As such, we decided that the urge to eat an unhealthy food would be an appropriate candidate for phenomenological comparison with hair pulling urges, since we also assumed that the satisfaction of unhealthy food urges is similar temporally to the satisfaction of hair pulling urges (i.e., durations of hair pulling and unhealthy food consumption are at least roughly comparable, such that there is not an excessively long time between initially giving in to the urge and ceasing the urge-related activity).

The goal of the current study was to compare the phenomenological features of hair pulling urges and urges to eat unhealthy foods across a number of dimensions. In this investigative context, two different groups of trichotillomania sufferers were of interest: those for whom unhealthy food urges were not a common problem and those for whom unhealthy food urges were a common problem. The former group was of interest because it was expected to report large and significant differences between the two kinds of urges across a number of dimensions. This would show that our survey method is able to identify such large and significant differences between urges when they exist. The second group, consisting of individuals with hair pulling urges as well as problematic unhealthy food urges, was of special interest. In particular, the current study sought to better understand the phenomenology of hair pulling urges by asking individuals with both kinds of urges to compare their hair pulling urges to their problematic urges to eat unhealthy foods. Participants were asked to compare the two urges across a number of dimensions frequently assessed in the trichotillomania phenomenology literature, including urge frequency, intensity, controllability, and associated affective states.

To identify the subset of participants for whom unhealthy food urges were a common problem, we developed a set of criteria questions analogous to the inclusion criteria for trichotillomania symptoms. Participants were asked whether they eat unhealthy foods on a regular basis, whether they have tried to stop or reduce their consumption of unhealthy foods, and whether their eating of unhealthy foods is distressing to them. Individuals who answered in the affirmative to these three unhealthy food criteria questions, and who also met the inclusion criteria for trichotillomania symptoms, were placed into the Unhealthy Food Positive (UF+) group. Individuals who only met the inclusion criteria for trichotillomania symptoms were placed into the “UF−” group.

In order to investigate the differences between hair pulling urges and unhealthy food urges in the UF+ and UF− groups, an internet-based survey was developed. The validity of internet survey methodologies in trichotillomania research has been addressed by Wetterneck et al. (2006), who found that participants recruited online reported similar demographics, trichotillomania severity, and other behavioral characteristics when compared to participants recruited in person at an educational research conference for trichotillomania (for a more general discussion of the validity of internet-based approaches, see Gosling et al., 2004).

In the present study, an internet-based survey was conducted in which both likert scale questions and free response questions were utilized. This approach allowed for the quantitative and qualitative comparison of the phenomenological characteristics of both hair pulling and unhealthy food urges. Results of the survey were expected to shed light on a number of issues. First, we sought to confirm that the UF– group would indeed report large and significant differences between the two types of urges, demonstrating the survey's ability to identify such large and significant differences when they exist. We then sought to explore the differences between the two urges, if any, identified by the UF+ group. In particular, we wished to identify specific urge dimensions on which the two urges differ and to characterize the magnitude of these differences. Finally, we hoped that the qualitative responses of participants would help to account for any differences between the two urges identified in this way.

## Methods

### Participants

This study was deemed exempt by the University of Michigan's Institutional Review Board. Participants were recruited through a link on the Trichotillomania Learning Center's research participation page (http://www.trich.org/involved/research-study.html). Participants were informed that the survey was anonymous and that no identifying information would be collected. A total of 511 responses were received, and 377 completed the survey. Of these, 208 participants met trichotillomania symptom inclusion criteria and were included in subsequent analyses. The trichotillomania symptom inclusion criteria for this study consisted of True-False questions asking participants to endorse DSM-5 trichotillomania criteria A, B, and C, as well as three further True-False questions designed to address criterion E (participants were asked whether they pull in response to certain delusions, e.g., “bugs crawling on the skin,” or whether they pull hair only for cosmetic reasons). Criterion D, which requires that the hair loss not be better explained by another medical condition (such as a dermatologic condition), could not be addressed. Table 1 displays the demographic and behavioral characteristics of the final sample. The data are displayed separately for the UF+ and UF- groups, which are described in *Analytic Strategy*.

**Table 1:** **Demographics and behavioral characteristics of the full participant sample, as well as of the Unhealthy Food Negative (UF-) subgroup and the Unhealthy Food Positive (UF+) subgroup**.

**Demographics**	**Full sample (*n* = 208)**	**UF− (n = 127)**	**UF+ (*n* = 81)**
**GENDER, % (n)**			
Female	95.2 (198)	92.9 (118)	98.8 (80)
Male	4.3 (9)	7.1 (9)	0.0 (0)
Other	0.5 (1)	0.0 (0)	1.2 (1)
**AGE**			
Mean (SD)	33.1 (12.4)	31.5 (12.1)	35.7 (13.1)
Range	18–67	18–67	18–65
**EDUCATION, % (n)**			
No high school	0.5 (1)	0.8 (1)	0.0 (0)
High school diploma or GED	18.3 (38)	20.5 (26)	14.8 (12)
Associate's or technical degree	21.2 (44)	18.1 (23)	25.9 (21)
Bachelor's degree	35.6 (74)	37.8 (48)	32.1 (26)
Master's degree	21.6 (45)	18.9 (24)	25.9 (21)
Doctoral or professional school degree	2.9 (6)	3.9 (5)	1.2 (1)
**ETHNICITY, % (n)**			
African-American or Black	2.9 (6)	2.4 (3)	3.7 (3)
Asian	1.9 (4)	1.6 (2)	2.5 (2)
Hispanic or Latin	3.4 (7)	3.9 (5)	2.5 (2)
White or Caucasian	82.7 (172)	80.3 (102)	86.4 (70)
Native American	1.0 (2)	0.8 (1)	1.2 (1)
Multiracial	6.3 (13)	7.9 (10)	3.7 (3)
Other	1.9 (4)	3.1 (4)	0.0 (0)
**ANNUAL INCOME, % (n)**			
Less than $10,000	18.8 (39)	24.4 (31)	9.9 (8)
$10,000 to $20,000	13.0 (27)	11.8 (15)	14.8 (12)
$20,000 to $30,000	16.3 (34)	16.5 (21)	16.0 (13)
$30,000 to $50,000	17.3 (36)	16.5 (21)	18.5 (15)
$50,000 to $75,000	18.8 (39)	15.7 (20)	23.5 (19)
More than $75,000	15.9 (33)	15.0 (19)	17.3 (14)
**MARITAL STATUS, % (n)**			
Single, never married	41.3 (86)	43.3 (55)	38.3 (31)
Living with partner	10.1 (21)	7.1 (9)	14.8 (12)
Married or domestic partnership	39.9 (83)	37.8 (48)	43.2 (35)
Divorced	3.4 (7)	3.1 (4)	3.7 (3)
Separated	0.0 (0)	0.0 (0)	0.0 (0)
Widowed	0.5 (1)	0.8 (1)	0.0 (0)
Other	4.8 (10)	7.9 (10)	0.0 (0)
**REPORTED SEEKING PROFESSIONAL HELP FOR A MENTAL HEALTH OR PSYCHOLOGICAL ISSUE, % (n)**			
Never	22.1 (46)	27.6 (35)	13.6 (11)
Yes, medical doctor–primary care physician	44.2 (92)	37.0 (47)	55.6 (45)
Yes, medical doctor–psychiatrist	47.6 (99)	40.9 (52)	58.0 (47)
Yes, psychologist	46.6 (97)	41.7 (53)	54.3 (44)
Yes, therapist or counselor	53.4 (111)	47.2 (60)	63.0 (51)
Other	10.1 (21)	8.7 (11)	12.3 (10)
**REPORTED HAVING BEEN FORMALLY DIAGNOSED WITH TRICHOTILLOMANIA BY A HEALTH CARE PROFESSIONAL, % (n)**			
Never	39.4 (82)	42.5 (54)	34.6 (28)
Yes, medical doctor–primary care physician	27.9 (58)	24.4 (31)	33.3 (27)
Yes, medical doctor–psychiatrist	34.6 (72)	29.1 (37)	43.2 (35)
Yes, psychologist	25.0 (52)	22.8 (29)	28.4 (23)
Yes, therapist or counselor	23.1 (48)	24.4 (31)	21.0 (17)
Other	4.3 (9)	2.4 (3)	7.4 (6)

### Survey development

An initial draft of the survey was created by the authors to compare hair pulling urges and urges to eat unhealthy foods along several dimensions. The survey was then reviewed by a trichotillomania specialist (a Master's level clinician with over 10 years of experience treating trichotillomania at a tertiary university clinic) and piloted on a patient with trichotillomania. The survey was revised based on the feedback received, and the final version was placed online. The full survey is available as Supplementary Data Sheet 1.

The final version of the survey consisted of several sections. The first section of the survey was devoted to questions about hair pulling and included trichotillomania symptom criteria questions, the Massachusetts General Hospital Hairpulling Scale (MGH-HPS, described below), as well as an assortment of likert-scale and free-response questions designed to assess a host of phenomenological characteristics, including affective correlates, resistibility of the urges, and cues for initiating and stopping hair pulling. The second section of the survey was devoted to questions about unhealthy foods and was designed to be symmetrical with the first section, using identical language wherever possible. An analogous version of the MGH-HPS was developed for this purpose. Additionally, a set of three unhealthy food criteria questions asked participants (in analogous fashion to the trichotillomania symptom criteria questions) whether they ate unhealthy food on a regular basis, whether they tried to stop or reduce their consumption of unhealthy food, and whether their eating of unhealthy food was distressing to them. In the third section of the survey, a series of head-to-head questions asked participants to directly compare phenomenological characteristics of the two urges in both likert-scale and free-response formats. The last section of the survey consisted of demographic questions.

### Instruments

#### Massachusetts general hospital hairpulling scale (MGH-HPS)

The MGH-HPS is a 7-item self-report measure of hair pulling severity over the last week. The individual items are rated from 0 to 4 and assess the following phenomenological characteristics: frequency of the urge, intensity of the urge, control of the urge, actual pulling, frequency of resistance attempts, frequency of resistance success, and distress associated with the urge. The psychometric properties of the MGH-HPS are good (O'Sullivan et al., 1995; Diefenbach et al., 2005; Keuthen et al., 2007), and this has been verified for an internet format (Keuthen et al., 2007).

#### The unhealthy food scale (UFS)

For the purposes of this study, an analogous version of the MGH-HPS was developed for unhealthy food urges, using identical language wherever possible. Although no psychometric data are available for this specially designed scale, our interest in this study was restricted to comparison of specific phenomenological characteristics between the two urges. Since participant ratings of the seven items were not summed to give a composite score, we did not need to worry about the interpretability of composite scores on the UFS. Instead, ratings from individual items on the MGH-HPS were compared to ratings from the analogous items on the UFS.

#### Analytic strategy

Individuals meeting trichotillomania symptom criteria were divided into two groups: participants meeting unhealthy food criteria questions (described in *Survey Development*) were placed in the “UF+ group,” and those failing to meet unhealthy food criteria were placed in the “UF- group.”

For each group, comparisons were then made between hair pulling urge characteristics and unhealthy food urge characteristics. Paired *t*-Tests were conducted to compare responses to individual items on the MGH-HPS and UFS. Repeated Measures ANOVA was performed to assess for the presence of certain emotions before, during, and after hair pulling or unhealthy food consumption. Bonferroni correction was applied where appropriate to correct for family-wise error rate. Finally, histograms of responses to the head to head comparison questions were developed.

Data from the free-response questions was analyzed using thematic analysis, a multi-stage methodology of coding qualitative data (Boyatzis, 1998; Saldaña, 2009). Participant responses to the qualitative questions formed the raw data. Statements made by participants about phenomenological characteristics were identified and analyzed for recurring patterns. Statements describing similar phenomenological processes were grouped together in categories and named using NVivo 10 software, a qualitative coding package. For instance, statements about hair pulling occurring automatically and without awareness were grouped together in a category called “Automatic, Unconscious Process.” Finally, related categories were grouped into themes to facilitate interpretation and the generation of a narrative explaining the data. A second researcher reviewed the categorization scheme and found the categorization scheme to be a persuasive classification of the data.

## Results

### Large differences found between the two urges in the UF− group, across multiple measures

The UF− group was asked to compare hair pulling and unhealthy food urges using a number of different measures. This allowed us to confirm that our survey instrument is capable of identifying large and significant differences between the two urges when they exist.

Results confirmed that for the UF− group, large and significant differences exist between the two urges. For example, ratings of hair pulling urge severity were significantly greater than ratings of unhealthy food urge severity on all urge properties measured, and the magnitudes of these differences were quite large (see Supplementary Figure 1. Differences between the urges were greater than 1.2 on a 4-point scale for 6 out of 7 urge properties measured). The complete data for the UF− group, including head-to-head comparisons of the two urges (Supplementary Figure 2) and affective changes over the course of urge satisfaction (Supplementary Figure 3, Supplementary Tables 2, 3) are available in the supplement.

Having provided this validation of our survey method, in the remainder of *Results*, we focus on findings for the UF+ group.

### Modest differences for only some urge properties in the UF+ group– MGH-HPS vs. UFS

**Figure 1:**
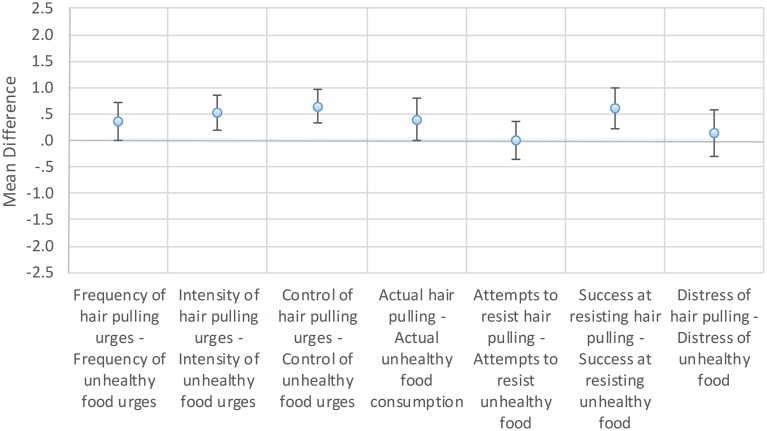
**Mean differences between hair-pulling urge properties and unhealthy food urge properties**. UF+ group: Mean differences between likert (0–4) scale responses on the Massachusetts General Hospital Hairpulling Scale (MGH-HPS) and the analogous Unhealthy Food Scale (UFS). Error bars show 99.3% confidence intervals to reflect the adjusted alpha level, which was set to 0.007 to correct for familywise error rate.

Seven phenomenological characteristics of hair pulling urges and unhealthy food urges were rated by participants from 0 to 4 (4 being the most severe) through their completion of the MGH-HPS and the UFS. Paired *t*-tests were conducted to compare participant ratings of analogous items on the MGH-HPS and UFS. The alpha level was set to 0.007 (0.05/7) to correct for familywise error rate. The results for the UF+ group are shown in Figure 1.

For the UF+ group, the differences between urge properties were modest. Four hair pulling urge characteristics were rated significantly higher than analogous unhealthy food urge characteristics. The mean differences between MGH-HPS and UFS ratings were 0.52 for urge intensity (*p* < 0.001, *d* = 0.46), 0.65 for the ability to control the urge (*p* < 0.001, *d* = 0.61), 0.40 for the amount of actual hair pulling or eating (*p* = 0.0064, *d* = 0.30), and 0.61 for success at resisting the urge (*p* < 0.001, *d* = 0.48). There were no significant differences for the other urge properties. The mean difference between MGH-HPS and UFS ratings was 0.36 for urge frequency (*p* = 0.0073), 0.00 for attempts to resist hair pulling or eating (*p* = 1.00), and 0.14 for distress associated with the urge (*p* = 0.379).

### In head-to-head comparisons, the UF+ group tended to rate hair pulling urges as more severe than urges to eat unhealthy foods

**Figure 2:**
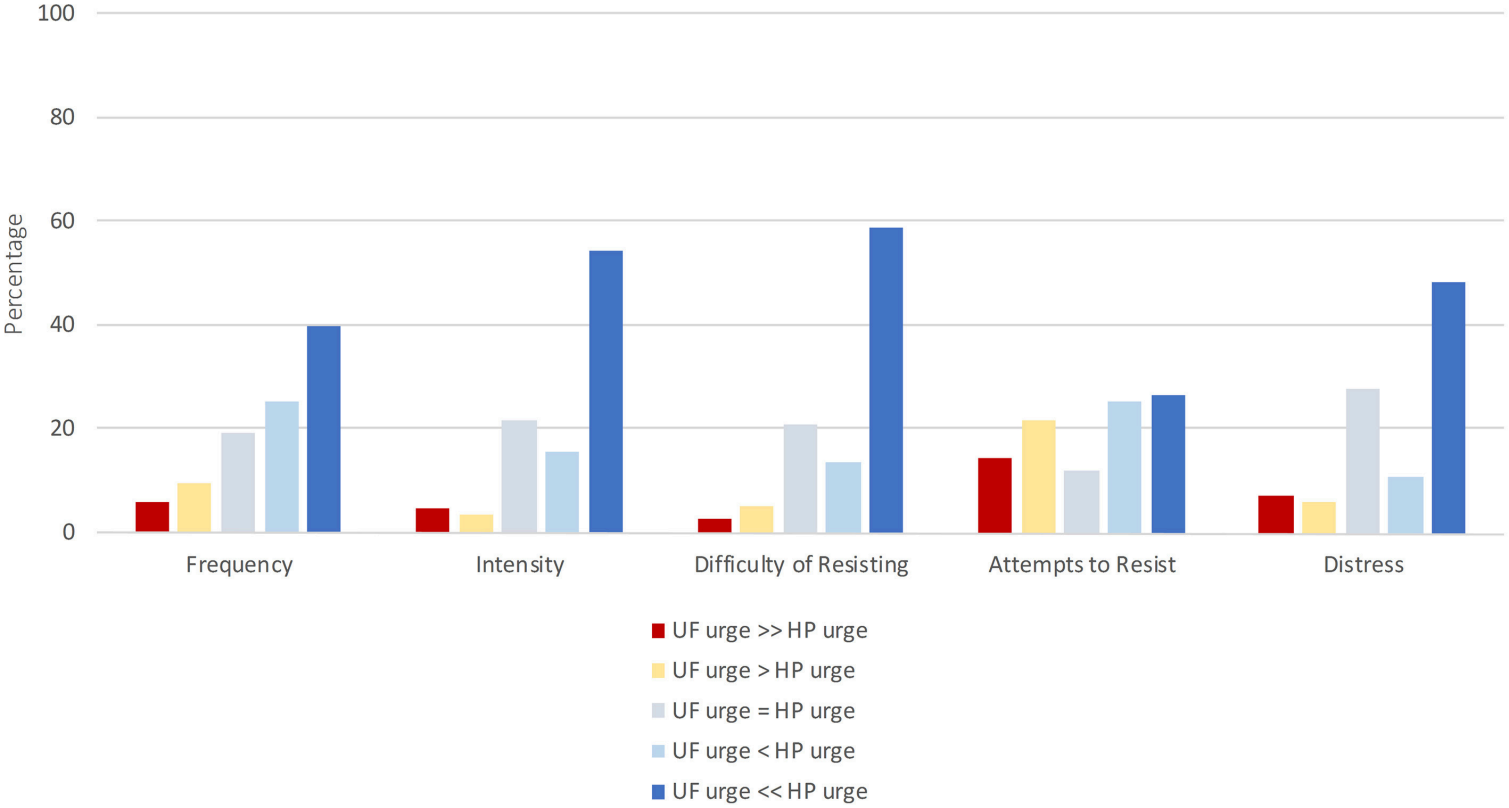
**Head to head comparisons of hair pulling and unhealthy food urges**. UF+ group: Histograms of responses to head-to-head questions asking participants to directly compare hair pulling and unhealthy food urges with respect to some property. For each urge property (frequency, intensity, difficulty of resisting, attempts to resist, and distress), participants indicated either that unhealthy food (UF) urges were equal in severity to hair pulling (HP) urges, or that one urge was “slightly” (>) more severe than the other, or that one urge was “much” more severe (>>) than the other.

To address the possibility that participants interpreted the 0–4 scales of the MGH-HPS and UFS scales differently, a series of head-to-head questions were devised asking participants to directly compare hair pulling and unhealthy food urges in the same question. The head-to-head questions were rated from 1 to 5, where a score of 3 indicated that the two urges were equal with respect to the phenomenological characteristic in question (e.g., intensity), a score above 3 indicated that the hair pulling urge was worse, and a score below 3 indicated that the unhealthy food urge was worse. Reverse coding was used every other question to combat framing effects. The response patterns to the five head-to-head questions are presented in histograms for the UF+ group in Figure 2.

The UF+ group tended to rate hair pulling urges as more severe than unhealthy food urges, such that for each urge property, 27–59% rated hair pulling urges as “much more severe” than unhealthy food urges. However, for each urge property, 27–47% of participants rated unhealthy food urges as equally severe, more severe, or much more severe than hair pulling urges.

### Affective changes are similar for the two urges in the UF+ group

**Figure 3:**
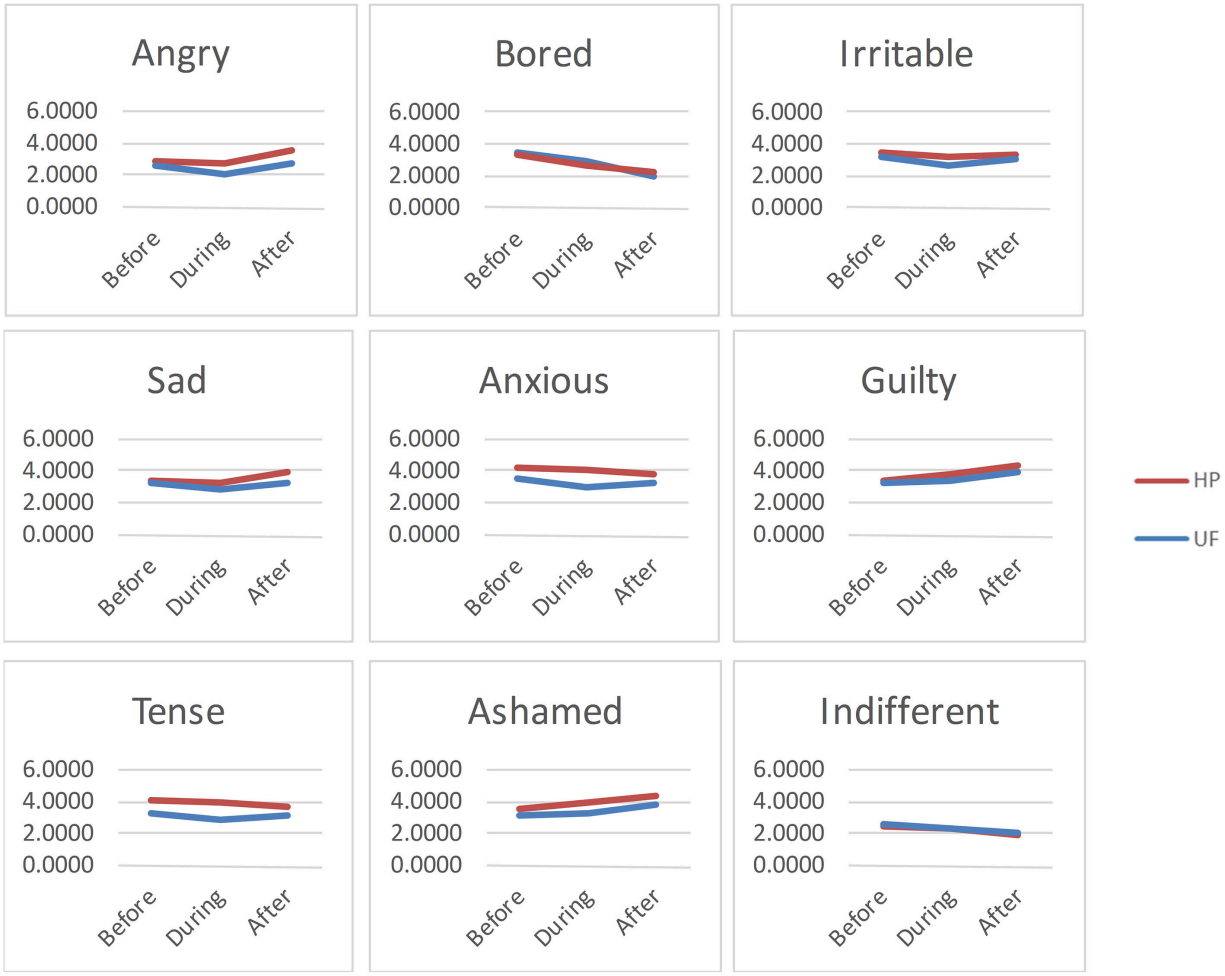
**Affective changes associated with urge satisfaction**. UF+ group: Plots of the changes in reported affect before, during, and after hair pulling (HP) or eating an unhealthy food (UF).

To examine the affective changes that accompany hair pulling and unhealthy food consumption, Repeated Measures ANOVA was performed to assess ratings of nine emotions or affective states before, during, and after either pulling or eating. The results for the UF+ group are shown in Table 2, and plots of affective states over time are shown for the UF+ group in Figure 3. The raw data for the reported affective states before, during, and after urge satisfaction are provided in the Supplement (Supplementary Table 1).

**Table 2:** Main effects of urge type, time, and urge type ^*^ time interaction for nine affective states.

**UF+**	**Urge type**			**Time**			**Urge** ^*^ **time**		
	**df1, df2**	**F**	**Sig**.	**df1, df2**	**F**	**Sig**.	**df1, df2**	**F**	**Sig**.
Angry	1, 76	14.509	0.000	2, 128.8	40.657	0.000	2, 136.1	5.093	0.010
Bored	1, 76	0.446	0.506	2, 152	98.340	0.000	2, 152	3.292	0.040
Irritable	1, 76	12.173	0.001	2, 152	12.495	0.000	1.7, 128.8	3.513	0.040
Sad	1, 76	11.542	0.001	2, 152	15.131	0.000	2, 152	5.553	0.005
Anxious	1, 76	39.686	0.000	2, 152	12.414	0.000	2, 152	3.286	0.040
Guilty	1, 76	8.375	0.005	2, 152	40.347	0.000	2, 152	0.925	0.399
Tense	1, 76	33.166	0.000	1.8, 133.5	7.824	0.001	2, 152	5.516	0.005
Ashamed	1, 76	10.835	0.002	2, 152	44.998	0.000	2, 152	1.239	0.293
Indifferent	1, 76	0.611	0.437	2, 152	20.288	0.000	2, 152	0.075	0.928

UF+ group: Results of a repeated measures ANOVA for nine different emotions examining the main effects of urge type (hair pulling or unhealthy food urges), time, and the urge type ^*^ time interaction. The Greenhouse-Geisser correction was applied where appropriate. (df = degrees of freedom).

For the UF+ group, there was a significant main effect of urge type (hair pulling or unhealthy food consumption) for anger, irritability, sadness, anxiousness, guilt, tenseness, and shame, but not for boredom or indifference (see Table 2). There was also a significant main effect of time for every emotion or affective state (see Table 2). However, there was a significant interaction between urge type and time for only 2 of 9 emotions: sadness and tenseness, but not for any of the other emotions or affective states (see Table 2). Sadness was found to be higher after hair pulling, but not unhealthy food consumption (see Figure 3, Supplementary Table 1). Tenseness decreased after both hair pulling and unhealthy food consumption, but the decline was slightly greater with hair pulling (see Figure 3, Supplementary Table 1).

### Qualitative questions—accounting for the differences between urges in the UF+ group

To help explain some of the significant differences between hair pulling and unhealthy food urges in the UF+ group, participants were asked a variety of questions asking them to compare hair pulling urges with unhealthy food urges in their own words. The question topics focused on whether the two urges feel different and on what brings the individual to stop either pulling or eating an unhealthy food. Here we present the major themes emerging from the analysis that highlight the differences and similarities between the phenomenology of hair pulling and unhealthy food urges.

### Hair pulling can be automatic and unconscious, but eating unhealthy food is a conscious process

A major theme emerging from the qualitative analysis involved the level of awareness at which hair pulling and unhealthy food consumption occur. Systematically and without prompting, participants commented about the automaticity and lack of awareness that often characterizes pulling, contrasted with the conscious awareness that accompanies unhealthy food consumption. These comments were made by participants in both the UF+ and UF− groups alike. Examples are shown in Table 3.

**Table 3:** **Examples of participant comments on the conscious or unconscious nature of eating unhealthy foods and hair pulling**.

**Conscious Process**	**Unconscious Process**
“I think the urge to eat unhealthy food is experienced on a more conscious level”	“My urge to pull is far less conscious”
“Eating is more per planned and some negotiation goes on first”	“The urge to pull hair is much more subconscious that eating”
“Eating is always conscious”	“Whereas the hair-pulling urge seems to be more subconscious - it sneaks up on me and is harder to resist”
“There is much more opportunity to think about those [unhealthy food] urges, and whether or not I'm making healthy choices”	“hairpulling urge involves a lot less conscious thinking than the urge to eat unhealthy”
“I am always aware when I am about to eat something bad for me”	“i am considerably less conscious of when I start pulling my hair or when I start again after trying to stop”

### Hair is close and available, but unhealthy foods are often less accessible, requiring thought and planning to acquire

A second major theme emerging from the analysis involved the spatiotemporal location of the object of hair pulling and unhealthy food urges. Without prompting, participants repeatedly mentioned the fact that hair is “right there,” close and accessible, that a person cannot get away from his or her hair, but that unhealthy food requires thought and planning to acquire, and that the individual can keep his or her distance from unhealthy food by not buying it at the store, leaving the room, etc. Once again, these comments were made by UF+ and UF- participants alike. Examples are shown in Table 4.

**Table 4:** **Examples of participant comments on whether the object of the urge (to eat an unhealthy food or pull hair) is removed, or close and accessible**.

**The object of the urge is removed**	**The object of the urge is close and accessible**
“Eating unhealthy food is a bit more of a process that requires more decision making, time and planning”	“But my eyebrows are always with me, so if I have an urge, it's always easy”
“For example, I don't keep unhealthy food in my house, so if I have an urge, it's much harder to satisfy”	“However pulling hair is “easier” since your hair is on your head and you have millions of it!”
“But I can keep food out of site and mind”	“BUT, unlike other addictions, I cannot remove myself from my hair”
“In contrast, I put more forethought into eating, and it takes actual thought, action and effort to get up and prepare or buy something to eat”	“However, my eyebrows are always there”
“The urge to eat is somewhat easier to control because I can physically remove myself from the situation—fill a plate at the potluck and physically sit down, and eat slow enough to finish at the same time as everyone else, or finish when the food is being put away, etc”	“Pulling hair, however, is something that can almost happen automatically and can also happen in any location, time of day, etc. because it involves another part of your body that is always accessible, whereas food is not”

### Hair pulling often involves a focused sensation or “itch” that is recurrent or persistent

A third major theme involved statements about itch-like or focused sensations that are recurrently or persistently on the individual's mind. Without prompting, participants made repeated mention of either the presence of focused sensations or itches, of the recurrent and persistent nature of hair pulling urges and sensations, or both. These statements were made by UF+ and UF− participants alike. Analogous statements were not made about unhealthy food urges. Examples are shown in Table 5.

**Table 5:** **Examples of participant comments on itches or focused sensations associated with hair pulling**.

**Itch or focused sensation associated with hair pulling**
“Often it starts with an unpleasant sensation in my scalp, like a tickle or itch or tingling or a tightness”
“Sometimes if I am consciously telling myself “don't touch your eyebrows” they start to itch - increasing the urge to touch them, which inevitably leads to pulling”
“It feels like…like i'm focusing on the area where i want to pull, even though i don't want to. Do you know that feeling you get when someone is staring at you? imagine them staring creepily at a specific area like your hand. well how your hand feels—that focused discomfort—is how the pull area feels”
“Urge to pull hair is more localized, like an itch to be relieved”
“When I get an urge to pull my hair, it feels as though all the hairs on my eyebrows are vibrating or tingling”

### Individuals stop pulling hair and eating unhealthy foods by similar mechanisms, but with different emphases

In response to questions about what brings it about that the individual stops pulling or eating, participants identified a similar set of mechanisms for the cessation of hair pulling and unhealthy food consumption. However, the relative emphasis of specific mechanisms sometimes differed for each activity. For instance, although a number of statements were made by participants about how they stop eating an unhealthy food upon suddenly realizing they have had too much, or are distracted by another person or activity, these types of awareness-increasing processes were especially emphasized in statements about hair pulling. Numerous statements were made about how hair pulling could be stopped by the presence of other people, by the sudden realization of how much hair had been pulled, or by having to move on to another activity, especially one that requires the use of the hands. Emotional and cognitive processes such as fear of the consequences of pulling or eating, telling oneself to stop, and reflecting on personal goals were cited as important in the cessation of both hair pulling and unhealthy food consumption. Both activities were also stopped after a feeling of satisfaction, such as after finding the “perfect hair” or satisfying a “craving” for an unhealthy food. Both activities were also stopped by physical limitations—individuals stopped pulling hair when their fingers, arms, or shoulders began to hurt excessively, and unhealthy food consumption was stopped upon feeling full or “sick.” Finally, individuals reported that for both activities, sometimes the individual only stopped because there was nothing left to pull (a bald spot in an area of interest) or eat (an unhealthy treat was wholly consumed). These statements were made by UF+ and UF- individuals alike. Examples are shown in Table 6.

**Table 6:** **Examples of participant comments on what brings it about that the individual stops eating an unhealthy food or pulling hair**.

**How to stop eating an unhealthy food**	**How to stop pulling hair**
“I stop eating because I realize I shouldn't be eating this much unhealthy food”	“Sometimes I stop if I hear that someone is coming”
“The thought of gaining weight”	“I usually pull when I am alone, so if someone comes into the room I might stop”
“I think about being accountable to my new structured routine of keeping a food diary”	“I usaually stop pulling after i realize how much hair i pulled”
“or just get to a point where I'm just ‘done’ such as with chips or a pint of ice cream”	“When I look down and there is a pile of hair I snap out of it”
“Craving satisfied”	“Change in activity. For example, if I have to write a paper, I pull while I'm writing. Then i finish the paper and I stop pulling. Or maybe I'm pulling “just because” but then someone calls me on the phone and I stop pulling”
“A full stomach”	“Sometimes I have to stop so I won't be late getting somewhere”
“Feelings of excess”	“I think about what pulling will do to affect me. Will I like what I see in the mirror after? The answer is always going to be no”
“The food usually makes me sick to my stomach, so I have to stop”	“Once I pull the offending lash or lashes causing the pain my eye feels much better and i am able to stop. But until I find the right lash I am unable to stop”
“OR the food makes me feel sick, so I stop eating”	“another reason I usually stop is because my hand or fingers are hurting from pulling for long periods of time”

## Discussion

While objective characteristics of hair pulling urges—including situational elicitors and typical responses—have been investigated in detail, the subjective phenomenology of these urges has not. The purpose of this study was to gain insight into the phenomenology of hair pulling urges by using a novel comparative methodology. In particular, we asked individuals with trichotillomania symptoms who also had problematic urges for unhealthy foods to systematically compare the two types of urges across multiple experiential dimensions. We found that while hair pulling urges were reported as more severe than unhealthy food urges across several dimensions, the differences were overall relatively moderate in size. Trichotillomania is sometimes seen as a puzzling disorder marked by seemingly irrational or incomprehensible urges. The results of the present study will help to fill in how those with the disorder experience their hair pulling urges.

Individuals with trichotillomania symptoms who do not report problematic urges to eat unhealthy foods (the UF− group) predictably rated hair pulling urges as significantly more severe than unhealthy food urges across all urge properties measured. This demonstrates the ability of our survey to identify substantial differences between urges when they exist. By contrast, the findings for the UF+ group call for a more nuanced interpretation.

The UF+ group tended to rate hair pulling urges as more severe than unhealthy food urges. However, the magnitude of the differences were perhaps not as great as one might have expected. First, differences showed up on only 4 out of 7 urge properties measured (intensity, controllability, actual pulling or eating, and success at resisting); for the other three urge properties (frequency, attempts to resist, and associated distress), no significant differences were observed. Second, examination of standardized effect sizes (Cohen's *d* values) showed that for the UF+ group, the differences that exist between the two urge types were small to moderate in size. Moreover, these effect sizes ranged from roughly a third to half as large as those reported in the UF− group. Third, the affective profiles associated with the two urge types were quite similar (we discuss this topic further below). These results are interesting because hair pulling urges are a manifestation of a potentially serious psychiatric condition, whereas we tend to think of unhealthy food urges as much more routine, everyday phenomena. Thus, one might have expected to see participants who experienced both types of urges to report dramatic differences in their respective phenomenologies. Sizable differences of this type, however, were not evident in our results.

Nevertheless, given that statistically reliable differences do exist between hair pulling urges and unhealthy food urges, it is natural to ask why such differences exist. The qualitative portion of the survey, which asked participants to directly compare hair pulling and unhealthy food urges in their own words, can potentially provide some insights. These qualitative responses highlighted certain important “situational” features of the two activities (hair pulling and unhealthy food consumption) which might help to explain differences between the two urges.

One factor participants repeatedly (and without prompting) brought up is the proximity of one's hair. This proximity of the urge object to the person likely contributes to the increased difficulty of controlling hair pulling urges, and thus the increased difficulty of successfully resisting such urges, as well the increased intensity these urges are perceived to have. Another factor repeatedly cited by participants is the way in which hair pulling can occur automatically, without the individual's awareness. This phenomenon, which is perhaps a direct byproduct of the proximity of one's hair, represents another process peculiar to hair pulling that appears to make pulling more difficult to control.

By contrast, participants emphasized that unhealthy food can be locked up in cupboards, left in the store, or otherwise removed from the person, requiring thought and planning to acquire. These features of unhealthy food acquisition are not properties of unhealthy food urges themselves, but represent features of the activity of unhealthy food acquisition that make the activity easier to manage and control. Natural bodily cues, such as feelings of fullness, represent further examples of ways in which processes external to the urge phenomenology can influence the control over the urge to eat an unhealthy food.

For the UF+ group, the temporal profile of affective changes associated with hair pulling and unhealthy food urges were remarkably similar, with 7 of 9 emotions exhibiting no statistically significant interactions. Temporal profiles did however differ for sadness and tenseness. A thorough account of these differences would require a deeper analysis than is possible here. One conjecture, however, is that increased sadness with hair pulling may reflect the social stigma associated with pulling, whereas the steeper decrease in tenseness may be explained by the proposal that hair pulling is used by individuals as a method of affect regulation (Diefenbach et al., 2008; Shusterman et al., 2009), a method which is easily available given the proximity of one's hair to the person.

This study has several limitations. First, participants were not asked about symptoms of binge-eating, or symptoms of eating disorders more generally. This is an important issue to address in future research on this subject. Second, considerations of survey length limited the number and scope of the different urge properties that could be measured. Future research comparing hair pulling urges to other urges, such as unhealthy food urges, should attempt to broaden the scope of urge properties measured, perhaps including some of the urge features mentioned by participants in the qualitative portion of the survey.

A third limitation of this study is that participants were recruited and assessed on the Trichotillomania Learning Center's website. It is possible that when participants are recruited and assessed through a trichotillomania-specific platform, they are relatively more predisposed to rate hair pulling urges as more severe than other urges (such as urges to eat unhealthy foods), which in turn may help to explain some of the observed differences between hair pulling urges and unhealthy food urges. If this hypothesis is right, however, then a main finding of our study—i.e., only moderate sized differences in the UF+ group between hair pulling urges and unhealthy food urges—should be seen as still more surprising; these relatively moderate differences were seen despite features of the study that tend to accentuate reporting of differences between the two types of urges.

Finally, although we believe our comparative method is able to yield unique insights into the phenomenology of hair pulling urges, this approach does face certain interpretive challenges. It is possible, for instance, that in isolating individuals with both types of urges (hair pulling and unhealthy food urges), we have identified a sample whose urges may be atypical. Individuals with trichotillomania symptoms, for example, may have more severe urges to eat unhealthy foods than the rest of the population. This is an important issue to address in future research. However, alternative approaches which compare urge phenomenology across different groups face their own difficulties, such as determining whether the different groups (e.g., hair pullers vs. binge eaters) rate the severity of their urges in comparable ways. The more direct comparison made possible by our comparative method avoids such difficulties.

It is hoped the knowledge gained from the present study will facilitate better understanding of the subjective experience of hair pulling urges by both clinicians and the lay public. Key findings from the present study are that hair pulling urges are rated as being only moderately more severe than problematic urges to eat unhealthy foods, and the differences that exist between the two urge types may at least in part be due to situational features of hair pulling, such as proximity and automaticity. This understanding may in turn help clinicians to achieve better rapport with individuals suffering from trichotillomania, as they will appreciate that resisting hair pulling may be more difficult than resisting an everyday urge. At the same time, the findings of the present study may facilitate lay understanding of the condition, and the comparison to an everyday urge may help to destigmatize what many people in the lay public may consider to be a puzzling condition. Our study suggests that hair pulling urges are not foreign processes that cannot be understood by those who have never experienced them. Hair pulling urges are likely more similar to unhealthy food urges than they are different, though there are some situational features of the activity of hair pulling that make the associated urges more difficult to resist.

Finally, the results of this study carry interesting implications for future research on the treatment of trichotillomania. For instance, techniques used to regulate everyday urges may have some efficacy for hair pulling urges. However, unique features of hair pulling may make hair pulling urges more resistant to intervention than everyday urges. By refining our understanding of just what these unique features of hair pulling are and to what extent they contribute to the phenomenology of hair pulling, future treatments may be able to more specifically target the features that make hair pulling urges more severe than everyday urges.

## Author contributions

CS and SM designed the study and wrote the survey. SM analyzed the data and wrote the first draft of the manuscript, and both CS and SM contributed to and have approved the final manuscript.

## Funding

CS's research was funded by NIH grants K23-AA-020297 and R01 MH107741. The funding source had no role in the study design, collection, analysis or interpretation of the data, writing the manuscript, or the decision to submit the paper for publication.

### Conflict of interest statement

The authors declare that the research was conducted in the absence of any commercial or financial relationships that could be construed as a potential conflict of interest.

## Supplementary material

Supplementary Figure 1 **Mean differences between hair-pulling urge properties and unhealthy food urge properties**. UF− group: Mean differences between likert (0–4) scale responses on the Massachusetts General Hospital Hairpulling Scale (MGH-HPS) and the analogous Unhealthy Food Scale (UFS). Error bars show 99.3% confidence intervals to reflect the adjusted alpha level, which was set to 0.007 to correct for familywise error rate.

Supplementary Figure 2 **Head to head comparisons of hair pulling and unhealthy food urges**. UF− group: Histograms of responses to head-to-head questions asking participants to directly compare hair pulling and unhealthy food urges with respect to some property. For each urge property (frequency, intensity, difficulty of resisting, attempts to resist, and distress), participants indicated either that unhealthy food (UF) urges were equal in severity to hair pulling (HP) urges, or that one urge was “slightly” (>) more severe than the other, or that one urge was “much” more severe (>>) than the other.

Supplementary Figure 3 **Affective changes associated with urge satisfaction**. UF– group: Plots of the changes in reported affect before, during, and after hair pulling (HP) or eating an unhealthy food (UF).

Supplementary Data Sheet 1 **Final version of the Hair Pulling and Unhealthy Food Phenomenology Survey used in this study**.

Supplementary Table 1 **UF+ group: Mean ratings (1–5) of affective states before, during, and after hair pulling (HP) or unhealthy food (UF) urge satisfaction**.

Supplementary Table 2 **UF− group: Mean ratings (1–5) of affective states before, during, and after hair pulling (HP) or unhealthy food (UF) urge satisfaction**.

Supplementary Table 3 **UF− group: Results of a repeated measures ANOVA for nine different emotions examining the main effects of urge type (hair pulling or unhealthy food urges), time, and the urge type ^*^ time interaction**. The Greenhouse-Geisser correction was applied where appropriate. (df, degrees of freedom).

## References

[B1] American Psychiatric Association (2013). Diagnostic and Statistical Manual of Mental Disorders, 5th Edn. Arlington, VA: American Psychiatric Publishing.

[B2] Boyatzis R. (1998). Transforming Qualitative Information: Thematic Analysis and Code Development. Thousand Oaks, CA: Sage Publications.

[B3] Christenson G. A. Mackenzie T. B. Mitchell J. E. (1991a). Characteristics of 60 adult chronic hair pullers. Am. J. Psychiatry 148, 365–370. 10.1176/ajp.148.3.365 1992841

[B4] Christenson G. A. Pyle R. L. Mitchell J. E. (1991b). Estimated lifetime prevalence of trichotillomania in college students. J. Clin. Psychiatry 52, 415–417. 1938977

[B5] Christenson G. A. Ristvedt S. L. Mackenzie T. B. (1993). Identification of trichotillomania cue profiles. Behav. Res. Therapy 31, 315–320. 10.1016/0005-7967(93)90030-X 8476406

[B6] Diefenbach G. J. Mouton-Odum S. Stanley M. A. (2002). Affective correlates of trichotillomania. Behav. Res. Therapy 40, 1305–1315. 10.1016/S0005-7967(02)00006-2 12384325

[B7] Diefenbach G. J. Tolin D. F. Crocetto J. Maltby N. Hannan S. (2005). Assessment of trichotillomania: a psychometric evaluation of hair-pulling scales. J. Psychopathol. Behav. Assess. 27, 169–178. 10.1007/s10862-005-0633-7

[B8] Diefenbach G. J. Tolin D. F. Meunier S. Worhunsky P. (2008). Emotion regulation and trichotillomania: a comparison of clinical and nonclinical hair pulling. J. Behav. Ther. Exp. Psychiatry 39, 32–41. 10.1016/j.jbtep.2006.09.002 17207769

[B9] Duke D. C. Bodzin D. K. Tavares P. Geffken G. R. Storch E. A. (2009a). The phenomenology of hairpulling in a community sample. J. Anx. Disord. 23, 1118–1125. 10.1016/j.janxdis.2009.07.015 19651487

[B10] Duke D. C. Keeley M. L. Ricketts E. J. Geffken G. R. Storch E. A. (2009b). The phenomenology of hairpulling in college students. J. Psychopathol. Behav. Assess. 32, 281–292. 10.1007/s10862-009-9150-4

[B11] du Toit P. L. van Kradenburg J. Niehaus D. J. Stein D. J. (2001). Characteristics and phenomenology of hair-pulling: an exploration of subtypes. Compr. Psychiatry 42, 247–256. 10.1053/comp.2001.23134 11349246

[B12] Flessner C. A. Conelea C. A. Woods D. W. Franklin M. E. Keuthen N. J. Cashin S. E. (2008). Styles of pulling in trichotillomania: exploring differences in symptom severity, phenomenology, and functional impact. Behav. Res. Ther. 46, 345–357. 10.1016/j.brat.2007.12.009 18249363

[B13] Gosling S. D. Vazire S. Srivastava S. John O. P. (2004). Should we trust web-based studies? A comparative analysis of six preconceptions about internet questionnaires. Am. Psychol. 59, 93–104. 10.1037/0003-066X.59.2.93 14992636

[B14] Grant J. E. Odlaug B. L. (2008). Clinical characteristics of trichotillomania with trichophagia. Compr. Psychiatry 49, 579–584. 10.1016/j.comppsych.2008.05.002 18970906 PMC2605948

[B15] Hofmann W. Vohs K. Baumeister R. (2012). What people desire, feel conflicted about, and try to resist in everyday life. Psychol. Sci. 23, 582–588. 10.1177/0956797612437426 22547657

[B16] Keuthen N. J. Flessner C. A. Woods D. W. Franklin M. E. Stein D. J. Cashin S. E. (2007). Factor analysis of the Massachusetts General Hospital Hairpulling Scale. J. Psychosom. Res. 62, 707–709. 10.1016/j.jpsychores.2006.12.003 17540230

[B17] Mansueto C. S. Stemberger R. M. Thomas A. M. Golomb R. G. (1997). Trichotillomania: a comprehensive behavioral model. Clin. Psychol. Rev. 17, 567–577. 10.1016/S0272-7358(97)00028-7 9260041

[B18] Martin D. Garske J. Davis M. (2000). Relation of the therapeutic alliance with outcome and other variables: a meta-analytic review. J. Consult. Clin. Psychol. 68, 438–450. 10.1037/0022-006X.68.3.438 10883561

[B19] O'Sullivan R. L. Keuthen N. J. Hayday C. F. Ricciardi J. N. Lynn Buttolph M. Jenike M. A.. (1995). The Massachusetts General Hospital (MGH) hairpulling scale: 2. reliability and validity. Psychother. Psychosom. 64, 146–148. 8657845 10.1159/000289004

[B20] Roberts S. O'Connor K. Bélanger C. (2013). Emotion regulation and other psychological models for body-focused repetitive behaviors. Clinical Psychology Review, 33, 745–762. 10.1016/j.cpr.2013.05.004 23792470

[B21] Saldaña J. (2009). The Coding Manual for Qualitative Researchers. London: Sage.

[B22] Shusterman A. Feld L. Baer L. Keuthen N. (2009). Affective regulation in trichotillomania: evidence from a large-scale internet survey. Behav. Res. Ther. 47, 637–644. 10.1016/j.brat.2009.04.004 19467648

[B23] Wetterneck C. T. Woods D. W. Norberg M. M. Begotka A. M. (2006). The social and economic impact of trichotillomania: results from two nonreferred samples. Behav. Inter. 21, 97–109. 10.1002/bin.211

[B24] Woods D. W. Flessner C. A. Franklin M. E. Keuthen N. J. Goodwin R. D. Stein D. J.. (2006). The Trichotillomania Impact Project (TIP): exploring phenomenology, functional impairment, and treatment utilization. J. Clin. Psychiatry 67, 1877–1888. 10.4088/JCP.v67n1207 17194265

