# Supplementary figures and images for: The Phenomenology of Hair Pulling Urges in Trichotillomania: A Comparative Approach
Source: Front Psychol. 2016 Feb 19;7:199. doi: 10.3389/fpsyg.2016.00199 (PMC4759292; doi:10.3389/fpsyg.2016.00199)

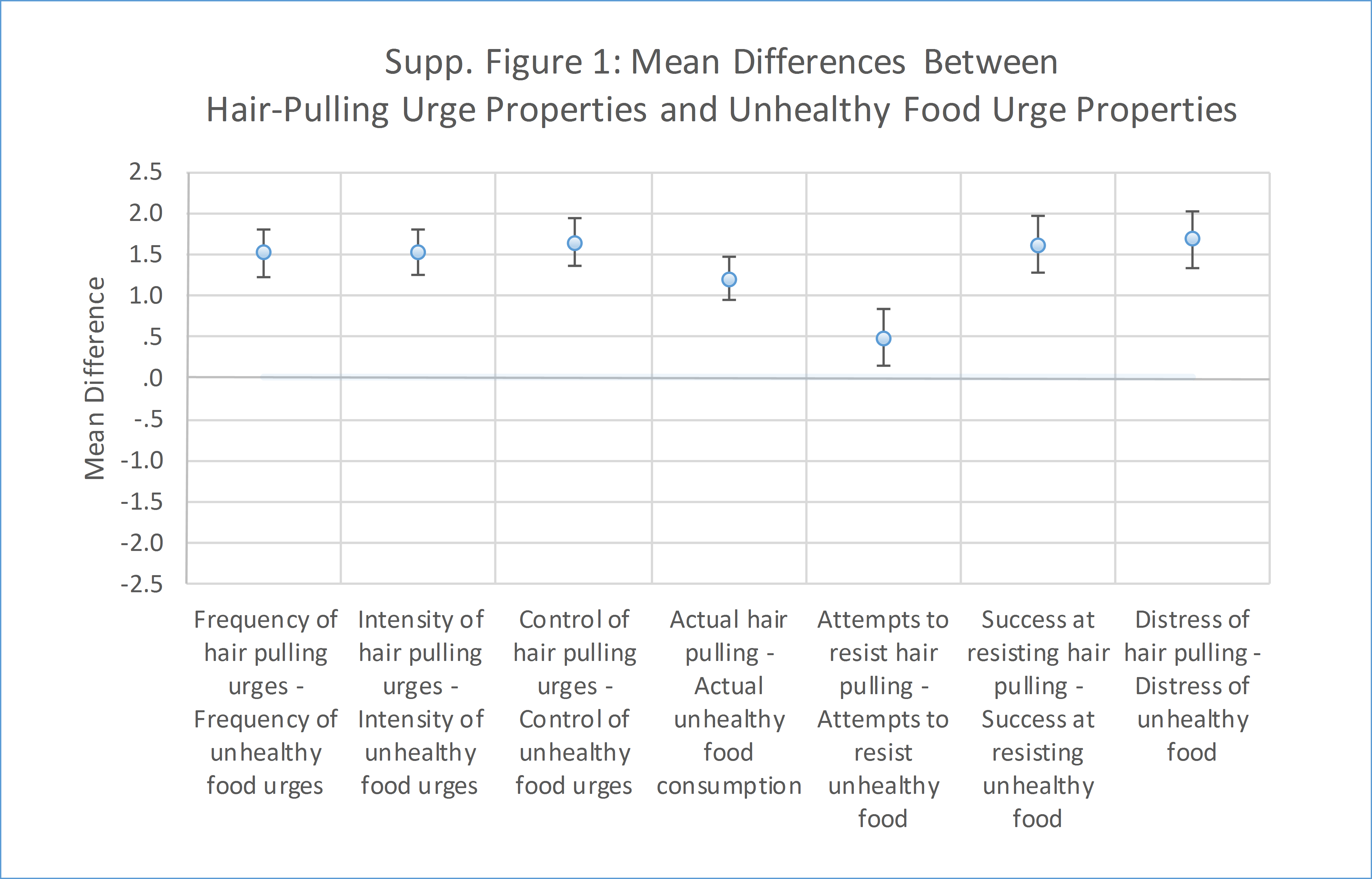

Supplement: Supplementary Figure 1 — Mean differences between hair-pulling urge properties and unhealthy food urge properties. UF− group: Mean differences between likert (0–4) scale responses on the Massachusetts General Hospital Hairpulling Scale (MGH-HPS) and the analogous Unhealthy Food Scale (UFS). Error bars show 99.3% confidence intervals to reflect the adjusted alpha level, which was set to 0.007 to correct for familywise error rate. [file Image1.TIF]

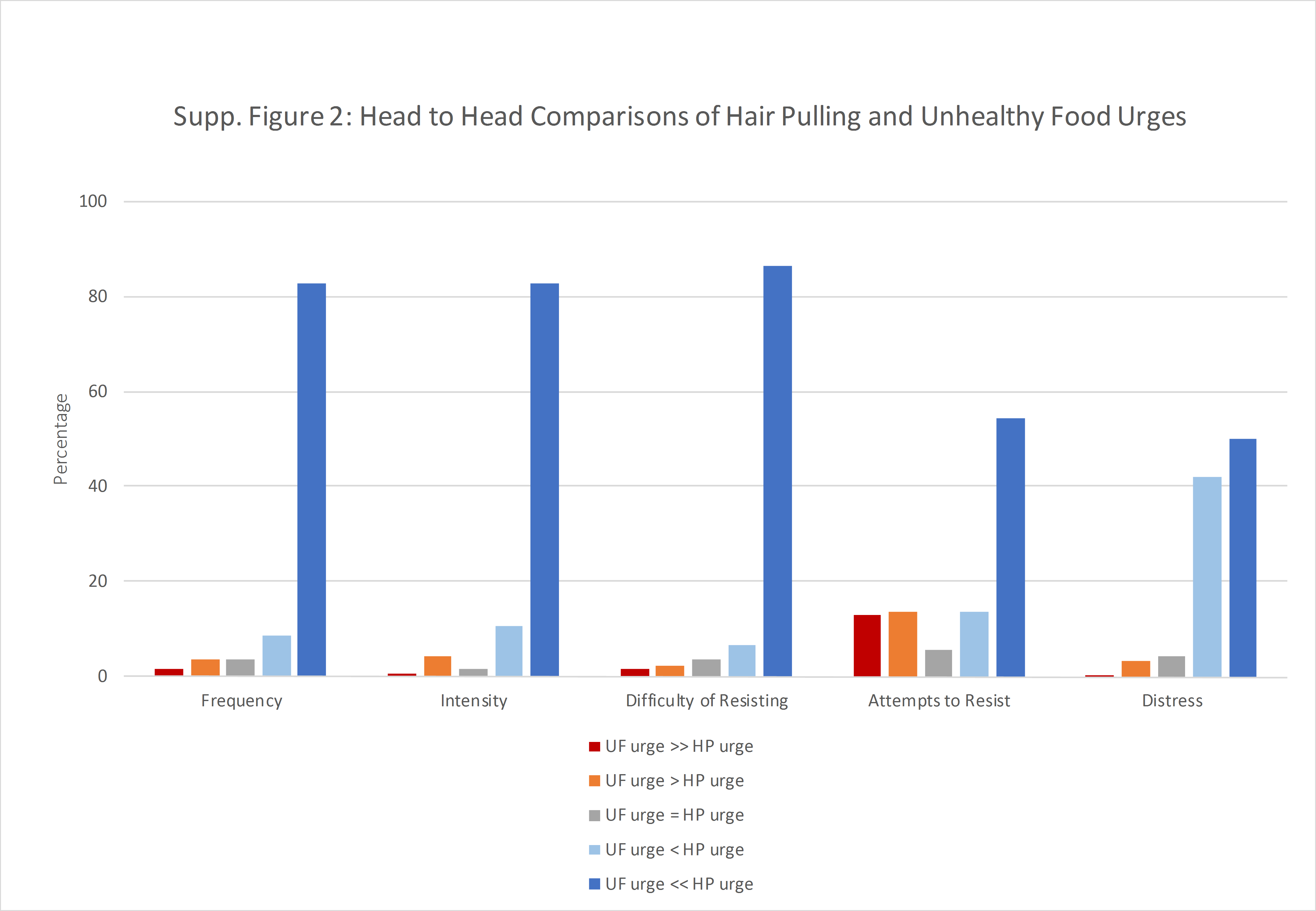

Supplement: Supplementary Figure 2 — Head to head comparisons of hair pulling and unhealthy food urges. UF− group: Histograms of responses to head-to-head questions asking participants to directly compare hair pulling and unhealthy food urges with respect to some property. For each urge property (frequency, intensity, difficulty of resisting, attempts to resist, and distress), participants indicated either that unhealthy food (UF) urges were equal in severity to hair pulling (HP) urges, or that one urge was “slightly” (>) more severe than the other, or that one urge was “much” more severe (>>) than the other. [file Image2.TIF]

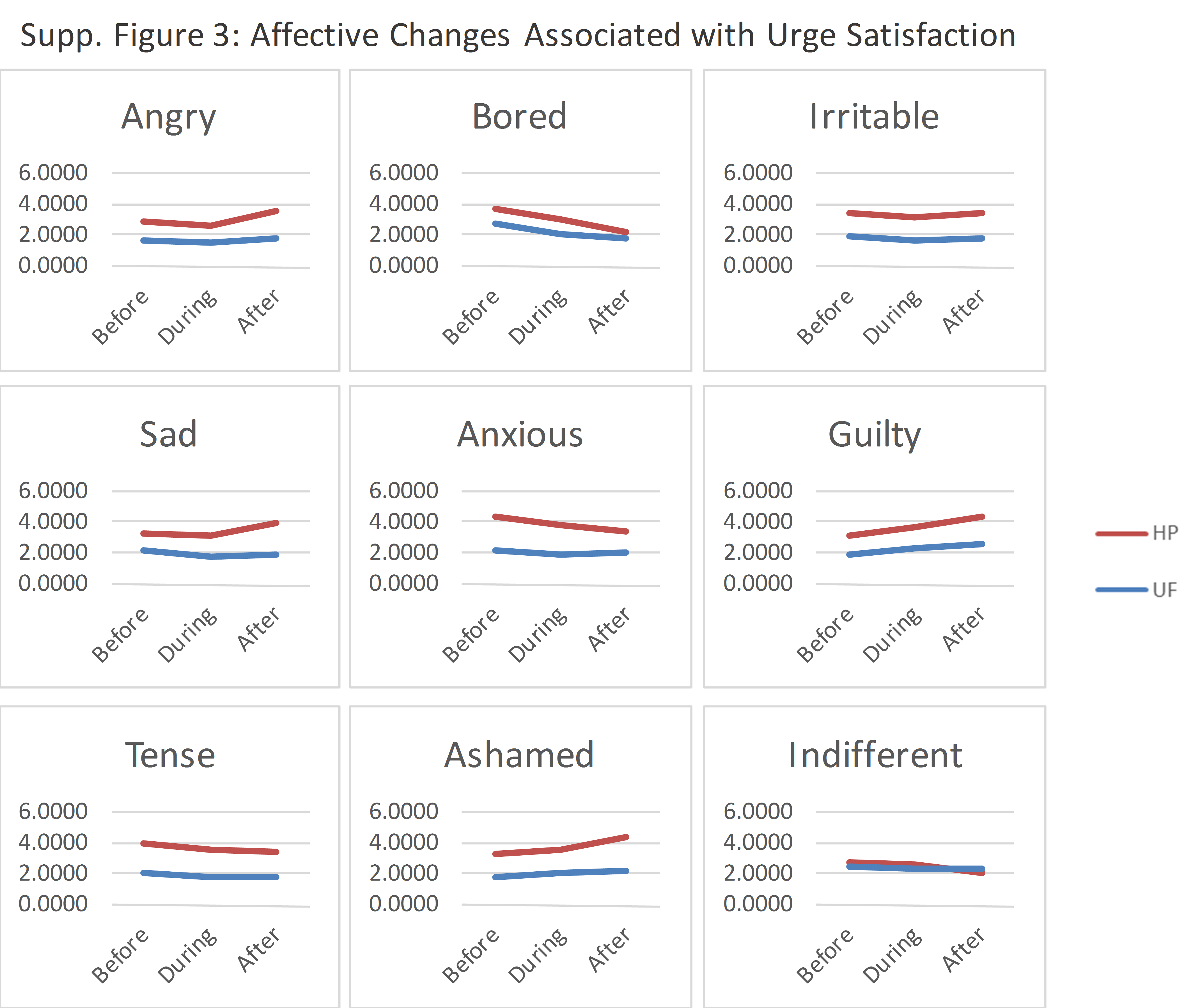

Supplement: Supplementary Figure 3 — Affective changes associated with urge satisfaction. UF– group: Plots of the changes in reported affect before, during, and after hair pulling (HP) or eating an unhealthy food (UF). [file Image3.TIF]
