# Supplementary material for: The Phenomenology of Hair Pulling Urges in Trichotillomania: A Comparative Approach
Source: Front Psychol. 2016 Feb 19;7:199. doi: 10.3389/fpsyg.2016.00199 (PMC4759292; doi:10.3389/fpsyg.2016.00199)
Supplement: Supplementary Data Sheet 1 — Final version of the Hair Pulling and Unhealthy Food Phenomenology Survey used in this study. [file DataSheet1.pdf]

Hair Pulling Urges

For this first set of questions, read each statement and decide whether it is true of your hair-pulling, on a scale of 0 to 9, where "0" means "not true of any of my hair-pulling" and "9" means "true of all of my hair pulling".

0 - Not true of any of my hair pulling
9- True of all of my hair pulling

	0	1	2	3	4	5	6	7	8	9
I pull my hair to get rid of an unpleasant urge, feeling, or thought.										
I pull my hair to control how I feel.										
I pull my hair because of something that has happened to me during the day.										
I have thoughts about wanting to pull my hair before I actually pull.										
I pull my hair when I am anxious or upset.										
I have a “strange” sensation just before I pull my hair.										
I pull my hair when I am experiencing a negative emotion, such as stress, anger, frustration, or sadness.										
I use tweezers or some other device other than my fingers to pull my hair.										
I intentionally start to pull my hair.										
I pull my hair while I am looking in the mirror.										
I don’t notice that I have pulled my hair until after it’s happened.										
I am usually not aware of pulling my hair during a pulling episode.										
I pull my hair when I am concentrating on another activity.										
I pull my hair when I am thinking about something unrelated to hair pulling.										
I am in an almost “trance-like” state when I pull my hair.										

For the questions in this section, pick the one statement which best describes your behaviors and/or feelings over the past week. If you have been having ups and downs, try to estimate an average for the past week. Be sure to read all the statements in each group before making your choice.

For the first three questions, rate only the urges to pull your hair.

Frequency of urges. On an average day, how often did you feel the urge to pull your hair?

- This week I felt no urges to pull my hair.
- This week I felt an occasional urge to pull my hair.
- This week I felt an urge to pull my hair often.
- This week I felt an urge to pull my hair very often.
- This week I felt near constant urges to pull my hair.

Intensity of urges. On an average day, how intense or “strong” were the urges to pull your hair?

- This week I did not feel any urges to pull my hair.
- This week I felt mild urges to pull my hair.
- This week I felt moderate urges to pull my hair.
- This week I felt severe urges to pull my hair.
- This week I felt extreme urges to pull my hair.

Ability to control the urges. On an average day, how much control do you have over the urges to pull your hair?

- This week I could always control the urges, or I did not feel any urges to pull my hair.
- This week I was always able to distract myself from the urges to pull my hair most of the time.
- This week I was able to distract myself from the urges to pull my hair some of the time.
- This week I was able to distract myself from the urges to pull my hair rarely.
- This week I was never able to distract myself from the urges to pull my hair.

For the next three questions, rate only the actual hairpulling.

Frequency of hairpulling. On an average day, how often did you actually pull your hair?

- This week I did not pull my hair.
- This week I pulled my hair occasionally.
- This week I pulled my hair often.
- This week I pulled my hair very often.
- This week I pulled my hair so often it felt like I was always doing it.

Attempts to resist hairpulling. On an average day, how often did you make an attempt to stop yourself from actually pulling your hair?

- This week I felt no urges to pull my hair.
- This week I tried to resist the urge to pull my hair almost all of the time.
- This week I tried to resist the urge to pull my hair some of the time.
- This week I tried to resist the urge to pull my hair rarely.
- This week I never tried to resist the urge to pull my hair.

Control over hairpulling. On an average day, How often were you successful at actually stopping yourself from pulling your hair?

- This week I did not pull my hair.
- This week I was able to resist pulling my hair almost all of the time.
- This week I was able to resist pulling my hair most of the time.
- This week I was able to resist pulling my hair some of the time.
- This week I was rarely able to resist pulling my hair..

For this question, rate the consequences of your hairpulling.

Associated distress. Hairpulling can make some people feel moody, “on edge,” or sad. During the past week, how uncomfortable did your hairpulling make you feel?

- This week I did not feel uncomfortable about my hairpulling.
- This week I felt vaguely uncomfortable about my hairpulling.
- This week I felt noticeably uncomfortable about my hairpulling.
- This week I felt significantly uncomfortable about my hairpulling.
- This week I felt intensely uncomfortable about my hairpulling.

In the next section, we will ask you to consider some basic statements about your hair-pulling. If the statement applies to you, click "True". If the statement does not apply to you, click "False".

I pull my hair out on a regular basis, resulting in either noticeable hair loss or a thinning of my hair (which may not be noticeable).

- True
- False

I have made repeated attempts to decrease or stop my hair-pulling.

- True
- False

The only reason I ever pull out hair is to improve my appearance.

- True
- False

I never, or almost never, pull my hair in response to voices that other people may not be able to hear.

- True
- False

I never, or almost never, pull my hair because I believe that something (e.g. an insect) is crawling on my skin.

- True
- False

My hair-pulling is distressing to me.

- True
- False

The next set of questions will ask you to indicate how your hair-pulling has affected your school or work, your social life, and your family life or home responsibilities.

The hair-pulling has affected your work /school work:

0 - not at all
1-3 - mildly
4-6 - moderately
7-9 - markedly
10 - extremely

0	1	2	3	4	5	6	7	8	9	10
○	○	○	○	○	○	○	○	○	○	○

The hair-pulling has disrupted your social life / leisure activities:

0 - not at all
1-3 - mildly
4-6 - moderately
7-9 - markedly
10 - extremely

0	1	2	3	4	5	6	7	8	9	10
○	○	○	○	○	○	○	○	○	○	○

The hair-pulling has disrupted your family life / home responsibilities:

0 - not at all
1-3 - mildly
4-6 - moderately
7-9 - markedly
10 - extremely

0	1	2	3	4	5	6	7	8	9	10
○	○	○	○	○	○	○	○	○	○	○

When people try to resist an urge, they sometimes succeed in resisting the urge, and they sometimes fail to resist the urge.

But sometimes, they manage to resist for a little while, and then they eventually give in to the urge. Let's call this "delaying" the urge.

For this question, estimate what percentage of the time you completely resist an urge, what percentage of the time you delay an urge, and what percentage of the time you fail to resist an urge at all.

	Never	Less than Half of the Time	Half of the time	More than Half of the Time	Always
When I try to resist an urge to pull my hair, I succeed in completely resisting the urge.	○	○	○	○	○
When I try to resist an urge to pull my hair, I manage to delay the urge, and then eventually I give in to the urge.	○	○	○	○	○
When I try to resist an urge to pull my hair, I fail to resist the urge at all.	○	○	○	○	○

Do you have any ideas about what it is that makes you start pulling hair on a certain occasion?

Feel free to identify any emotions, thoughts, or events that you think might cause you to start pulling your hair.

Below is a list of many different emotions. For this question, indicate how often you feel the emotion while having the urge to pull your hair, but *before* actually starting to pull your hair.

When I have the urge to pull my hair, I feel...

	Never	Rarely	Sometimes	Often	Always
Angry					
Bored					
Irritatable					
Sad					
Anxious					
Guilty					
Tense					
Ashamed					
Indifferent					

Below is a list of many different emotions. For this question, indicate how often you feel the emotion while you are actually pulling your hair.

While I am actually pulling my hair, I feel...

	Never	Rarely	Sometimes	Often	Always
Angry					
Bored					
Irritatable					
Sad					
Anxious					
Guilty					
Tense					
Ashamed					
Indifferent					

Once you have started pulling your hair on some occasion, you eventually stop pulling. Do you have any ideas about what happens to make you eventually stop pulling?

Feel free to identify any emotions, thoughts, or events that you think might cause you to stop pulling your hair once you've started.

Below is a list of many different emotions. For this question, indicate how often you feel the emotion after you have finished pulling your hair.

After I have finished pulling my hair, I feel...

	Never	Rarely	Sometimes	Often	Always
Angry					
Bored					
Irritatable					
Sad					
Anxious					
Guilty					
Tense					
Ashamed					
Indifferent					

Unhealthy Food Urges

In the next part of this survey, we will ask you questions about "everyday" urges. We will focus in particular on the urge to eat an unhealthy food.

For this next set of questions, read each statement and decide whether it is true of your urges to eat unhealthy food, on a scale of 0 to 9, where "0" means "not true of any of my eating of unhealthy food" and "9" means "true of all of my eating of unhealthy food".

0 - Not true of any of my eating of unhealthy food
9- True of all of my eating of unhealthy food

	0	1	2	3	4	5	6	7	8	9
I eat unhealthy food to get rid of an unpleasant urge, feeling, or thought.										
I eat unhealthy food to control how I feel.										
I eat unhealthy food because of something that has happened to me during the day.										
I have thoughts about wanting to eat an unhealthy food before I actually eat.										
I eat unhealthy food when I am anxious or upset.										
I have a “strange” sensation just before I eat an unhealthy food.										
I eat unhealthy food when I am experiencing a negative emotion, such as stress, anger, frustration, or sadness.										
I use a utensil or some other device other than my fingers to eat an unhealthy food.										
I intentionally start to eat an unhealthy food.										
I eat unhealthy food while I am looking in the mirror.										
I don’t notice that I have eaten an unhealthy food until after it’s happened.										
I am usually not aware of eating unhealthy food during an eating episode.										
I eat unhealthy food when I am concentrating on another activity.										
I eat unhealthy food when I am thinking about something unrelated to eating.										
I am in an almost “trance-like” state when I eat an unhealthy food.										

For each question, pick the one statement in that group which best describes your behaviors and/or feelings over the past week. If you have been having ups and downs, try to estimate an average for the past week. Be sure to read all the statements in each group before making your choice.

For the next three questions, rate only your urges to eat an unhealthy food.

Frequency of urges. On an average day, how often did you feel the urge to eat an unhealthy food?

- This week I felt no urges to eat an unhealthy food.
- This week I felt an occasional urge to eat an unhealthy food.
- This week I felt an urge to eat an unhealthy food often.
- This week I felt an urge to eat an unhealthy food very often.
- This week I felt near constant urges to eat unhealthy food.

Intensity of urges. On an average day, how intense or "strong" were the urges to eat an unhealthy food?

- This week I did not feel any urges to eat an unhealthy food.
- This week I felt mild urges to eat an unhealthy food.
- This week I felt moderate urges to eat an unhealthy food.
- This week I felt severe urges to eat an unhealthy food.
- This week I felt extreme urges to eat an unhealthy food.

Ability to control the urges. On an average day, how much control do you have over the urges to eat an unhealthy food?

- This week I could always control the urges, or I did not feel any urges to eat an unhealthy food.
- This week I was able to distract myself from the urges to eat an unhealthy food most of the time.
- This week I was able to distract myself from the urges to eat an unhealthy food some of the time.
- This week I was able to distract myself from the urges to eat an unhealthy food rarely.
- This week I was never able to distract myself from the urges to eat an unhealthy food.

For the next three questions, rate only the actual eating of unhealthy food.

Frequency of eating unhealthy food. On an average day, how often did you actually eat an unhealthy food?

- This week I did not eat an unhealthy food.
- This week I ate an unhealthy food occasionally
- This week I ate an unhealthy food often.
- This week I ate an unhealthy food very often.
- This week I ate an unhealthy food so often it felt like I was always doing it.

Attempts to resist eating an unhealthy food. On an average day, how often did you make an attempt to stop yourself from actually eating an unhealthy food?

- This week I felt no urges to eat an unhealthy food.
- This week I tried to resist the urge to eat an unhealthy food almost all of the time.
- This week I tried to resist the urge to eat an unhealthy food some of the time.
- This week I tried to resist the urge to eat an unhealthy food rarely.
- This week I never tried to resist the urge to eat an unhealthy food.

Control over eating unhealthy food. On an average day, how often were you successful at actually stopping yourself from eating an unhealthy food?

- This week I did not eat an unhealthy food.
- This week I was able to resist eating an unhealthy food almost all of the time.
- This week I was able to resist eating an unhealthy food most of the time.
- This week I was able to resist eating an unhealthy food some of the time.
- This week I was rarely able to resist eating an unhealthy food.

For the last question, rate the consequences of your eating unhealthy food.

Associated distress. During the past week, how uncomfortable did your eating unhealthy food make you feel?

- This week I did not feel uncomfortable about my eating unhealthy food.
- This week I felt vaguely uncomfortable about my eating unhealthy food.
- This week I felt noticeably uncomfortable about my eating unhealthy food.
- This week I felt significantly uncomfortable about my eating unhealthy food.
- This week I felt intensely uncomfortable about my eating unhealthy food.

The next couple of questions we will ask you to consider some basic statements about eating unhealthy food. If the statement applies to you, click "True". If the statement does not apply to you, click "False".

I eat unhealthy food on a somewhat regular basis.

- True
- False

I have tried in the past to decrease or stop my eating of unhealthy food.

- True
- False

My eating unhealthy food is distressing to me.

- True
- False

When people try to resist an urge, they sometimes succeed in resisting the urge, and they sometimes fail to resist the urge.

But sometimes, they manage to resist for a little while, and then they eventually give in to the urge. Let's call this "delaying" the urge.

For this question, estimate what percentage of the time you completely resist an urge, what percentage of the time you delay an urge, and what percentage of the time you fail to resist an urge at all.

	Never	Less than Half the Time	Half the Time	More than Half the Time	Always
When I try to resist an urge to eat an unhealthy food, I succeed in completely resisting the urge.	○	○	○	○	○
When I try to resist an urge to eat an unhealthy food, I manage to delay the urge, and then eventually I give in to the urge.	○	○	○	○	○
When I try to resist an urge to eat an unhealthy food, I fail to resist the urge at all.	○	○	○	○	○

Do you have any ideas about what it is that makes you start eating an unhealthy food on a certain occasion?

Feel free to identify any emotions, thoughts, or events that you think might cause you to start eating an unhealthy food.

Below is a list of many different emotions. For this question, indicate how often you feel the emotion while having the urge to eat an unhealthy food, but *before* actually starting to eat it.

When I have the urge to eat an unhealthy food, I feel...

	Never	Rarely	Sometimes	Often	Always
Angry	○	○	○	○	○
Bored	○	○	○	○	○
Irritatable	○	○	○	○	○
Sad	○	○	○	○	○
Anxious	○	○	○	○	○
Guilty	○	○	○	○	○
Tense	○	○	○	○	○
Ashamed	○	○	○	○	○
Indifferent	○	○	○	○	○

Below is a list of many different emotions. For this question, indicate how often you feel the emotion while you are actually eating an unhealthy food.

While I am actually eating an unhealthy food, I feel...

	Never	Rarely	Sometimes	Often	Always
Angry	○	○	○	○	○
Bored	○	○	○	○	○
Irritatable	○	○	○	○	○
Sad	○	○	○	○	○
Anxious	○	○	○	○	○
Guilty	○	○	○	○	○
Tense	○	○	○	○	○
Ashamed	○	○	○	○	○
Indifferent	○	○	○	○	○

Once you have started eating an unhealthy food on some occasion, you eventually stop eating it. Do you have any ideas about what happens to make you eventually stop eating the unhealthy food?

Feel free to identify any emotions, thoughts, or events that you think might cause you to stop eating an unhealthy food once you've started.

Below is a list of many different emotions. For this question, indicate how often you feel the emotion after you have finished eating an unhealthy food.

After I have finished eating an unhealthy food, I feel...

	Never	Rarely	Sometimes	Often	Always
Angry	○	○	○	○	○
Bored	○	○	○	○	○
Irritatable	○	○	○	○	○
Sad	○	○	○	○	○
Anxious	○	○	○	○	○
Guilty	○	○	○	○	○
Tense	○	○	○	○	○
Ashamed	○	○	○	○	○
Indifferent	○	○	○	○	○

Comparison Questions

In this section, we will ask you to directly compare your hair-pulling urges to your urges to eat an unhealthy food.
For each question, think of the TYPICAL example of each kind of urge.

Which urge do you experience more often (hair-pulling urge or urge to eat an unhealthy food)?

- My urge to eat an unhealthy food is MUCH more frequent than my urge to pull hair.
- My urge to eat an unhealthy food is SOMEWHAT more frequent than my urge to pull hair
- The two urges are equally frequent.
- My urge to pull hair is SOMEWHAT more frequent than my urge to eat an unhealthy food.
- My urge to pull hair is MUCH more frequent than my urge to eat an unhealthy food.

Which urge is "stronger", or more intense?

- My urge to pull hair is MUCH more intense than my urge to eat an unhealthy food.
- My urge to pull hair is SOMEWHAT more intense than my urge to eat an unhealthy food.
- The two urges are equally intense.
- My urge to eat an unhealthy food is SOMEWHAT more intense than my urge to pull hair.
- My urge to eat an unhealthy food is MUCH more intense than my urge to pull hair.

Which urge is more difficult to resist?

- My urge to eat an unhealthy food is MUCH more difficult to resist than my urge to pull hair.
- My urge to eat an unhealthy food is SOMEWHAT more difficult to resist than my urge to pull hair.
- The two urges are equally difficult to resist.
- My urge to pull hair is SOMEWHAT more difficult to resist than my urge to eat an unhealthy food.
- My urge to pull hair is MUCH more difficult to resist than my urge to eat an unhealthy food.

Which urge do you try to resist more often?

- I try to resist my urge to pull hair MUCH more often than I try to resist my urge to eat an unhealthy food.
- I try to resist my urge to pull hair SOMEWHAT more often than I try to resist my urge to eat an unhealthy food.
- I try to resist the two urges with equal frequency.
- I try to resist my urge to eat an unhealthy food SOMEWHAT more often than I try to resist my urge to pull hair.
- I try to resist my urge to eat an unhealthy food MUCH more often than I try to resist my urge to pull hair.

Which urge is more distressing to you?

- My urges to eat an unhealthy food are MUCH more distressing to me than my urges to pull hair.
- My urges to eat an unhealthy food are SOMEWHAT more distressing to me than my urges to pull hair.
- The two urges are equally distressing.
- My urges to pull hair are SOMEWHAT more distressing to me than my urges to eat an unhealthy food.
- My urges to pull hair are MUCH more distressing to me than my urges to eat an unhealthy food.

Take a moment now to think about what it is like to feel an urge to pull your hair. Focus on what the urge feels like before you have actually started pulling.

Some people believe that an urge to pull hair feels very different from an everyday urge, such as an urge to eat something unhealthy.

What do you think about this? Do you agree or disagree? Please explain.

Some people think that hair-pulling urges are irresistible (or almost irresistible), unlike everyday urges. What do you think about that? Do you agree or disagree? Please explain.

Some people think that urges to eat unhealthy food are irresistible (or almost irresistible). What do you think about that? Do you agree or disagree? Please explain.

Satisfying an urge sometimes makes us feel better. Satisfying an urge can make us feel more relaxed, it can distract us from other things that are bothering us, and it can sometimes make us happy.

Compare pulling your hair to eating an unhealthy food. Which one seems to make you feel better?

The questions in this survey were designed to help us gain a better understanding of how a hair-pulling urge *feels* in comparison to an everyday urge like the urge to eat an unhealthy food.

Do you think there is anything else we should know about what hair-pulling urges feel like, or how they compare to everyday urges? Feel free to discuss anything that you think is relevant to this question. If you have nothing to add, you may skip this question.

Demographic Questions

Please answer the following background questions. These questions will help us identify the general characteristics of the people who participated in this survey. Your responses will be completely anonymous.

What is your gender?

- Male
- Female
- Other (please specify below)

Please type in your age.

0 Age

What is the highest level of education or level of school that you have completed?

- No high school completed
- High school diploma or GED
- Associate's degree or technical school degree
- Bachelor's degree
- Master's degree
- Doctoral or professional school degree

What is your ethnicity?

- African-American or Black
- Asian
- Hispanic or Latin
- White or Caucasian
- Native American
- Mutliracial
- Other (please specify below)

What is your annual income?

- Less than $10,000
- $10,000 to $20,000
- $20,000 to $30,000
- $30,000 to $50,000
- $50,000 to $75,000
- More than $75,000

What is your marital status?

- Single, never married
- Living with partner
- Married or domestic partnership
- Divorced
- Separated
- Widowed
- Other (please specify below)

How many children do you have?

- 0
- 1
- 2
- 3
- 4+

Have you ever sought professional help for a mental health or psychological issue? If so, which health professionals did you see? Please check all that apply.

- [ ] I have never sought professional help for a mental health or psychological issue.
- [ ] Yes, Medical doctor - primary care physician (family doctor, internist, etc.)
- [ ] Yes, Medical doctor - psychiatrist
- [ ] Yes, Psychologist
- [ ] Yes, Therapist or counselor
- [ ] Other (please specify)

Have you ever been formally diagnosed with trichotillomania by a health care professional? Please check all that apply.

- [ ] I have never been formally diagnosed with trichotillomania by a health care professional.
- [ ] Yes, by a medical doctor - primary care physician (family doctor, internist, etc.)
- [ ] Yes, by a medical doctor - psychiatrist
- [ ] Yes, by a psychologist
- [ ] Yes, by a therapist or counselor
- [ ] Yes, by another professional, not listed (please specify)

Have you ever been formally diagnosed with a mental disorder other than trichotillomania?

- ( ) Yes
- ( ) No

Please identify the mental disorders with which you have been diagnosed by a health care professional (primary care physician, psychiatrist, psychologist, therapist, or counselor). Please check all that apply.

- ☐ Attention-deficit hyperactivity disorder (ADHD)
- ☐ Autism
- ☐ Panic disorder
- ☐ Obsessive-compulsive disorder (OCD)
- ☐ Generalized anxiety disorder (GAD)
- ☐ Post-traumatic stress disorder (PTSD)
- ☐ Social anxiety disorder (social phobia)
- ☐ Agoraphobia
- ☐ Specific phobia
- ☐ Major depressive disorder (MDD)
- ☐ Bipolar disorder
- ☐ Dysthymic disorder
- ☐ Eating disorder
- ☐ Personality disorder
- ☐ Body dysmorphic disorder
- ☐ hoarding disorder
- ☐ Skin-picking (excoriation) disorder
- ☐ Schizophrenia
- ☐ Other (please specify) ____________

Survey Powered By Qualtrics
