# Supplementary material for: The Phenomenology of Hair Pulling Urges in Trichotillomania: A Comparative Approach
Source: Front Psychol. 2016 Feb 19;7:199. doi: 10.3389/fpsyg.2016.00199 (PMC4759292; doi:10.3389/fpsyg.2016.00199)
Supplement: Supplementary Table 1 — UF+ group: Mean ratings (1–5) of affective states before, during, and after hair pulling (HP) or unhealthy food (UF) urge satisfaction. [file Table1.DOCX]

| **Supp. Table 1: Mean Ratings of Affective States Associated with Urge Satisfaction** | | | | |
| --- | --- | --- | --- | --- |
| **UF+** | **Urge** | **Before** | **During** | **After** |
| Angry | HP | 2.7922 | 2.7403 | 3.4675 |
|  | UF | 2.6234 | 2.0909 | 2.7662 |
| Bored | HP | 3.4286 | 2.8571 | 2.0130 |
|  | UF | 3.2597 | 2.6104 | 2.1948 |
| Irritable | HP | 3.4156 | 3.2338 | 3.3506 |
|  | UF | 3.1948 | 2.5844 | 2.9870 |
| Sad | HP | 3.3377 | 3.2468 | 3.8701 |
|  | UF | 3.2338 | 2.8052 | 3.1818 |
| Anxious | HP | 4.2078 | 4.0000 | 3.8052 |
|  | UF | 3.4935 | 2.9870 | 3.2078 |
| Guilty | HP | 3.4156 | 3.7532 | 4.3377 |
|  | UF | 3.2078 | 3.3247 | 3.9610 |
| Tense | HP | 4.0519 | 3.9091 | 3.6234 |
|  | UF | 3.2857 | 2.8182 | 3.0779 |
| Ashamed | HP | 3.4545 | 3.8831 | 4.3377 |
|  | UF | 3.1429 | 3.3117 | 3.8182 |
| Indifferent | HP | 2.4416 | 2.2727 | 1.9221 |
|  | UF | 2.5844 | 2.3506 | 2.0390 |
