# Supplementary material for: The Phenomenology of Hair Pulling Urges in Trichotillomania: A Comparative Approach
Source: Front Psychol. 2016 Feb 19;7:199. doi: 10.3389/fpsyg.2016.00199 (PMC4759292; doi:10.3389/fpsyg.2016.00199)
Supplement: Supplementary Table 2 — UF− group: Mean ratings (1–5) of affective states before, during, and after hair pulling (HP) or unhealthy food (UF) urge satisfaction. [file Table2.DOCX]

| **Supp. Table 2: Mean Ratings of Affective States Associated with Urge Satisfaction** | | | | |
| --- | --- | --- | --- | --- |
| **UF-** | **Urge** | **Before** | **During** | **After** |
| Angry | HP | 2.7874 | 2.5984 | 3.5039 |
|  | UF | 1.6142 | 1.4961 | 1.7480 |
| Bored | HP | 3.6220 | 2.9685 | 2.1339 |
|  | UF | 2.7008 | 2.0630 | 1.7559 |
| Irritable | HP | 3.4409 | 3.1654 | 3.3386 |
|  | UF | 1.9449 | 1.6614 | 1.7874 |
| Sad | HP | 3.1811 | 3.0315 | 3.8583 |
|  | UF | 2.0945 | 1.7559 | 1.9055 |
| Anxious | HP | 4.2677 | 3.7874 | 3.4252 |
|  | UF | 2.1575 | 1.8425 | 1.9843 |
| Guilty | HP | 3.1024 | 3.5906 | 4.3386 |
|  | UF | 1.9213 | 2.2677 | 2.4961 |
| Tense | HP | 3.9528 | 3.5591 | 3.4488 |
|  | UF | 1.9685 | 1.6850 | 1.7953 |
| Ashamed | HP | 3.1969 | 3.5669 | 4.3465 |
|  | UF | 1.6929 | 1.9685 | 2.1417 |
| Indifferent | HP | 2.6929 | 2.5433 | 2.0157 |
|  | UF | 2.4331 | 2.3228 | 2.2677 |
