# Supplementary material for: The Phenomenology of Hair Pulling Urges in Trichotillomania: A Comparative Approach
Source: Front Psychol. 2016 Feb 19;7:199. doi: 10.3389/fpsyg.2016.00199 (PMC4759292; doi:10.3389/fpsyg.2016.00199)
Supplement: Supplementary Table 3 — UF− group: Results of a repeated measures ANOVA for nine different emotions examining the main effects of urge type (hair pulling or unhealthy food urges), time, and the urge type * time interaction. The Greenhouse-Geisser correction was applied where appropriate. (df, degrees of freedom). [file Table3.DOCX]

| Supp. Table 3: Main Effects of Urge Type, Time, and Urge Type * Time Interaction for Nine Affective States | | | | | | | | | |
| --- | --- | --- | --- | --- | --- | --- | --- | --- | --- |
| UF- |  | Urge Type |  |  | Time |  |  | Urge * Time |  |
|  | df1, df2 | F | Sig. | df1, df2 | F | Sig. | df1, df2 | F | Sig. |
| Angry | 1, 126 | 141.665 | .000 | 1.8, 223.9 | 40.232 | .000 | 1.7, 220.0 | 15.630 | .000 |
| Bored | 1, 126 | 47.294 | .000 | 2, 252 | 150.577 | .000 | 2, 252 | 10.864 | .000 |
| Irritable | 1, 126 | 187.748 | .000 | 2, 252 | 10.068 | .000 | 2, 252 | .099 | .906 |
| Sad | 1, 126 | 149.163 | .000 | 2, 252 | 31.074 | .000 | 2, 252 | 31.118 | .000 |
| Anxious | 1, 126 | 287.420 | .000 | 2, 252 | 32.924 | .000 | 2, 252 | 17.227 | .000 |
| Guilty | 1, 126 | 145.988 | .000 | 2, 252 | 84.287 | .000 | 1.8, 231.0 | 10.686 | .000 |
| Tense | 1, 126 | 254.028 | .000 | 2, 252 | 18.173 | .000 | 2, 252 | 3.232 | .041 |
| Ashamed | 1, 126 | 202.377 | .000 | 2, 252 | 84.202 | .000 | 1.7, 208.2 | 15.294 | .000 |
| Indifferent | 1, 126 | .328 | .568 | 2, 252 | 22.336 | .000 | 2, 252 | 7.787 | .001 |
